# Identification of SCAMP2 as a regulator of NOTCH signaling in cisplatin resistance through a novel prognostic model for bladder cancer

**DOI:** 10.3389/fimmu.2025.1573412

**Published:** 2025-05-08

**Authors:** Longjun Cai, Shaoqi Zhang, Fangfang Zheng, Furong Ji, Jin Wang, Long Shi, Liu Chao, Xiangyu Wang, Jianjun Zhang, Zhiyong Chen

**Affiliations:** ^1^ Department of Urology, The Affiliated Suqian Hospital of Xuzhou Medical University, Nanjing Drum-Tower Hospital Group Suqian Hospital, Suqian, Jiangsu, China; ^2^ School of Public Health, Suzhou Medicine College of Soochow University, Suzhou, Jiangsu, China; ^3^ Department of Urology, The Affiliated Shuyang Hospital of Xuzhou Medical University, Suqian, China

**Keywords:** cisplatin sensitivity, prognostic model, bladder cancer, *SCAMP2*, notch pathway

## Abstract

**Introduction:**

Bladder cancer remains a major challenge in clinical oncology, particularly due to the development of platinum resistance, which severely impacts patient prognosis. Despite numerous attempts to create effective prognostic models, their clinical applicability has often been limited.

**Methods:**

In this study, we utilized a robust statistical approach, LASSO-COX regression analysis, to develop a novel prognostic model for bladder cancer based on cisplatin sensitivity-related genes (CSRGs). The model was validated using both the TCGA-BLCA dataset and an independent validation set, GSE32894. Additionally, we employed various *in vitro* assays, including CCK-8 and EdU assays for cell proliferation, transwell assays for migration, and flow cytometry for apoptosis analysis, to investigate the biological function of the identified genes.

**Results:**

Our prognostic model demonstrated superior predictive performance, with high AUC values. SCAMP2 was identified as a critical gene with elevated expression in bladder cancer, showing strong correlation with sensitivity to multiple anti-cancer drugs, including cisplatin. Further functional assays revealed that SCAMP2 mediates drug resistance in bladder cancer cells via the NOTCH signaling pathway. Additionally, *in vivo* experiments showed that SCAMP2 overexpression significantly enhanced cisplatin sensitivity in bladder cancer tissues.

**Discussion:**

These findings underscore the potential of CSRGs, particularly SCAMP2, as critical biomarkers for bladder cancer prognosis. The identification of SCAMP2 as a regulator of NOTCH signaling in cisplatin resistance offers new insights into the molecular mechanisms of chemotherapy resistance and suggests potential therapeutic targets for overcoming drug resistance. Our model could guide personalized treatment strategies and improve bladder cancer patient outcomes.

## 1 Introduction

Bladder cancer is a complex disease, ranks as the tenth most common malignancy worldwide, with approximately 573,000 new cases and about 213,000 deaths annually (1). Of these cases, around 75% are diagnosed as non-muscle invasive bladder cancer (NMIBC), with the remainder classified as muscle-invasive bladder cancer (MIBC) (2). Patients with MIBC face a relatively poor prognosis, with the standard treatment being radical cystectomy (3). Despite aggressive treatment, the five-year survival rate for patients with MIBC remains only between 20% and 40%, and nearly half of the cases progress to a metastatic state within three years of diagnosis.

As a cornerstone in the treatment of bladder cancer, cisplatin plays a pivotal role both in neoadjuvant therapy and in the treatment of metastatic disease (4, 5). To reduce side effects while maintaining anti-tumor efficacy, several other platinum compounds have been developed, including carboplatin, oxaliplatin, and nedaplatin (6–9). However, the importance of cisplatin in the treatment of bladder cancer remains significant. Regrettably, only about 35% of patients with metastatic bladder cancer initially respond to cisplatin chemotherapy, and most of those initially sensitive to treatment will ultimately develop resistance to cisplatin (10, 11). Recent research has revealed various biological mechanisms of cisplatin resistance, including alterations in drug transport and metabolism, enhanced DNA repair mechanisms, aberrations in cell cycle regulation, and inhibition of apoptotic pathways (12). Cisplatin resistance is one of the major factors contributing to the poor prognosis of patients with metastatic bladder cancer.

The Genomics of Drug Sensitivity in Cancer (GDSC) database (www.cancerRxgene.org) is the largest public repository providing extensive data on cancer cell drug sensitivity and molecular markers of drug response (13). In this study, leveraging the GDSC database and the “Oncopredict” package (14), we calculated cisplatin sensitivity scores for bladder cancer (BLCA) samples in the The Cancer Genome Atlas (TCGA) database and identified cisplatin sensitivity-related genes (CSRGs) through correlation analysis. The aim of this study is to construct a risk prediction model for bladder cancer patients using these CSRGs, assessing its feasibility, robustness, and biological value for clinical application. The significance of this research is to facilitate the development of personalized treatment strategies, potentially increasing patients’ chemotherapy responsiveness.

## 2 Materials and methods

### 2.1 Data collection

The BLCA data of 407 patients, including 19 normal tissues adjacent to cancer tissues, 407 tumor tissues and corresponding clinical information, were retrieved from The Cancer Genome Map (TCGA) database. The expression profile and clinical results are open and accessible. To validate the prognostic model based on the TCGA BLCA cohort, another BLCA dataset (GSE32894) was retrieved from the Gene Expression Omnibus (GEO) database as an external validation dataset. The GSE32894 (15) dataset contains gene expression data and prognosis information for 224 primary BLCA samples.

### 2.2 Drug sensitivity analysis

The Genomics of Drug Sensitivity in Cancer (GDSC) database was developed by the Sanger Research Institute to collect data on the sensitivity and response of tumor cells to drugs (13). “OncoPredict” was used to calculate the drug sensitivity of each sample in the training and validation datasets based on the GDSC V2.0 database (14). Then, genes related to cisplatin sensitivity (CSRGs) were identified through correlation analysis, with the correlation threshold being the absolute value of the correlation coefficient > 0.3, p< 0.05.

### 2.3 Prognostic model construction and validation

The chi-square test was used to analyze the differences between the training set, the internal test set and the total dataset in terms of different Clinicopathological characteristics. The univariate Cox model was used to study the relationship between continuous expression levels of CRGs and OS. The risk ratio (HR) and P value from the univariate Cox regression analysis were used to identify candidate survival-related CRGs. CRGs with an HR > 1 were considered risky CRGs, and those with an HR< 1 were defined as protective CRGs. CRGs that met the criterion of a P value<0.05 were identified as survival-related CRGs and further included in LASSO and multivariate Cox regression analyses to construct a prognostic model. The risk score for each BLCA patient was calculated based on the expression of CRGs (Exp_i_) and Cox coefficients (coef_i_) $\text{Risk score}\;=\overset{n}{\underset{i=1}{\sum }}Exp_{i}\times coef_{i}$ . All patients in each dataset were divided into high- or low-risk groups according to the median value. K–M plots were generated to evaluate patient survival in each dataset between the high- and low-risk groups. Moreover, multivariate Cox regression analysis was performed to estimate whether the risk score was independent of clinicopathological features. To investigate the performance of the prognostic model in predicting BLCA patient outcomes, the area under the curve (AUC) of the ROC curve (AUC) was calculated. In addition, the expression of each CRGS in the model and its correlation with clinicopathological features were also analyzed.

All analyses were performed with R software (version 4.3.1) and the corresponding fundamental package. The “care” package was used to randomly divide the patients into two datasets at a ratio of 6:4 according to their survival status, which were used as training sets and internal test sets, respectively. The “glmnet” package was used for LASSO regression model analysis. In addition, the “survival” and “survminer” packages were used to perform univariate and multivariate Cox analyses and to generate Kaplan–Meier plots. The “TimeROC” package was used to generate the time-dependent receiver operating characteristic (ROC) curve, and the “survivalROC” package was used to calculate the area under the curve (AUC). Nomogram plots were generated with the “rms” package.

### 2.4 Enrichment analysis

Based on the correlation analysis between the risk score and all mRNAs, gene set enrichment analysis (GSEA) was further performed by using the “ClusterProfiler” package of R software (version 4.3.1).

In addition, the differentially expressed genes (DEGs) between the low and high groups were identified based on the R package “limma” with the thresholds of log(fold change) >1 and P value< 0.05. The DEGs were further input into the DAVID online tool (https://david.ncifcrf.gov/) for pathway and biological process enrichment.

### 2.5 Correlation analysis

To further explore the biological role and clinical significance of the DRG prognostic model, correlation analysis was performed between the risk score and the expression of oncogenes, tumor mutation burden (TMB), immune regulatory gene expression, immune cell infiltration and tumor immune dysfunction and exclusion (TIDE) score. Correlation analysis was performed with the Spearman method based on the “psych” package.

The oncogenes were extracted from the ONGene database (http://www.ongene.bioinfo-minzhao.org) (16). A total of 73 immunomodulatory genes (IMGs) (17) were extracted from previous studies. The immune cell infiltration score was calculated by using the XCELL algorithm (18). Moreover, the TIDE score, dysfunction score and exclusion score of each patient in the datasets were predicted using the TIDE online tool (http://tide.dfci.harvard.edu/) following standard procedures (19).

### 2.6 shRNA and overexpression plasmid construction

*SCAMP2* shRNA sequences were designed according to BLOCK-iT™ RNAi Designer (https://rnaidesigner.thermofisher.com/rnaiexpress), and the annealed double-stranded shRNA was cloned and inserted into the pGreen vector. After testing the knockdown efficiency of several candidate shRNAs, the sequence 5’-GGGTCACTATGGAGTTCAAAG-3’ targeting *SCAMP2* and the sequence 5’-GCAGCTGAAATATCCTAAACT-3’ targeting FTO were selected for subsequent experiments. A scrambled nonspecific control shRNA (shNC) was also cloned and inserted into the same vector and used as a negative control. For overexpression, the full-length coding sequence of *SCAMP2* was amplified and cloned and inserted into the pCDH plasmid.

### 2.7 Cell culture and transfection

The human lung cancer cell lines A549 and H1299 were purchased from the American Type Culture Collection (ATCC). All cells were cultured in DMEM (Thermo Fisher Scientific, Inc.) supplemented with 10% FBS (Thermo Fischer Scientific, Inc.) at 37°C in the presence of 5% CO2.

GC cells were seeded in 6-well plates in each well and grown for 24 h. Then, the cells were transfected with 2.5 μg of shSCAMP2 or shNC using Lipofectamine 6000 reagent (Beyotime, China) following the manufacturer’s protocol.

### 2.8 Cell proliferation assay

For cell proliferation, lung cancer cells were initially seeded into 6-well plates. These cells were then incubated with 10 μM EdU for 2 hours. Next, the cells were stabilized with 4% paraformaldehyde and permeabilized using 0.3% Triton X-100, a process conducted in a PBS environment. A subsequent step involved incubating the cells with a click reaction solution, a product provided by the Beyotime Institute of Biotechnology in China. Within a 24-hour timeframe, images of the cells were obtained using an inverted fluorescence microscope, and the resulting data were analyzed with the assistance of NIH ImageJ software (version 1.8.0).

### 2.9 Cell migration assay

In terms of the cell migration assay, cells from each group were methodically placed in the upper chambers of each Transwell membrane (Corning, Inc., USA). Next, 1 ml of medium without FBS and 2 ml of complete medium were added to the bottom chamber. After a 24-hour incubation period at 37°C in an environment with 5% CO2, the cells were stabilized in methanol and stained with 0.5% crystal violet for 30 minutes. The final stage involved washing the cells in the upper chamber with phosphate-buffered saline (PBS, provided by Gibco, USA) three times. The cells were then imaged using a microscope and evaluated with NIH ImageJ software (version 1.8.0).

### 2.10 Cell apoptosis assay

For the apoptosis assay, cells were seeded in six-well plates at a density of approximately 1 × 10^5^ cells per well. After 24 hours of incubation, cells were transfected with shSCAMP2. Following transfection, cells were harvested and washed twice with cold phosphate-buffered saline (PBS). Apoptosis was assessed using the *PI*/*AnnexinV*−*FITC* Apoptosis Detection Kit according to the manufacturer’s instructions. Briefly, cells were resuspended in 1X binding buffer and then stained with PI and Annexin V-FITC for 15 minutes at room temperature in the dark. After staining, cells were analyzed by flow cytometry within 1 hour to quantify the percentage of apoptotic cells.

### 2.11 Quantitative Real-Time PCR (qPCR)

Total RNA was extracted from cells using the TRIzol reagent (Invitrogen, USA) according to the manufacturer’s protocol. cDNA was synthesized from 1 µg of total RNA using the PrimeScript RT Reagent Kit (Takara, Japan). qPCR was performed using the SYBR Green PCR Master Mix (Applied Biosystems, USA) on an ABI 7500 Real-Time PCR System (Applied Biosystems, USA). The primer sequences for MAML1, MAML2, MAML3, NOTCH2, NOTCH3, and ACTB (used as an internal control) are listed in **Supplementary Table 1**. The relative expression levels of target genes were calculated using the 2^-ΔΔCt method and normalized to ACTB.

### 2.12 Western blot

Cells were lysed in RIPA buffer (Thermo Scientific, USA) supplemented with protease and phosphatase inhibitors (Roche, Switzerland). Protein concentrations were determined using the BCA Protein Assay Kit (Pierce, USA). Equal amounts of protein (30 µg) were separated by SDS-PAGE and transferred to PVDF membranes (Millipore, USA). Membranes were blocked with 5% non-fat milk in TBST for 1 hour at room temperature and subsequently incubated overnight at 4°C with primary antibodies against SCAMP2, NOTCH2, and MAML1 (all from Proteintech, USA), and ACTB (used as a loading control, Sigma-Aldrich, USA). After washing, membranes were incubated with HRP-conjugated secondary antibodies (Jackson ImmunoResearch, USA) for 1 hour at room temperature. Protein bands were visualized using the ECL detection system (Thermo Scientific, USA) and quantified by ImageJ software (NIH, USA).

### 2.13 Animal model and drug treatment

All animal experiments adhered to institutional animal ethics guidelines. Male nude mice aged 6–8 weeks were used in the experiments. The mice were acclimated to the environment for at least one week prior to the experiments, under conditions of a 12-hour light/dark cycle, 22°C, and 40-60% humidity, with free access to standard chow and water. To establish the bladder cancer model, T24 cells were stably transfected with plasmids overexpressing SCAMP2 (SCAMP2 group) or empty vector plasmids (control group). The transfection was performed using Lipofectamine 2000 according to the manufacturer’s protocol. After 24–48 hours, the cells were selected with 2μg/ml puromycin to ensure the establishment of stable transfectants. The successful overexpression of SCAMP2 was confirmed by quantitative PCR. Following selection and verification of SCAMP2 overexpression levels, these cells were subcutaneously injected into the right hind flank of the mice to establish the tumor model. Approximately 7 days post-inoculation, when the tumor volume reached around 100 mm³, cisplatin treatment was initiated. Both the SCAMP2 overexpression group and the control group received cisplatin treatment at a dose of 10 mg/kg (administered intraperitoneally) once a week for 3 weeks. During the experiment, tumor volume was measured regularly to assess tumor growth. Tumor volume was measured using a caliper, recording the length (L) and width (W) of the tumors, and calculated using the formula V = (L × W²)/2. Tumor growth curves were plotted based on weekly volume measurements. At the end of the experiment, mice were euthanized, and tumors were excised, weighed using a balance, and subjected to quantitative comparison.

### 2.14 Immunohistochemistry (IHC)

To detect the impact of SCAMP2 overexpression on related protein expression, tumor tissues were fixed in formalin and embedded in paraffin. Sections of 4 μm thickness were prepared and subjected to immunohistochemical staining using specific antibodies against SCAMP2, CDH2, NOTCH2, Ki67, and CDH1. The immunohistochemistry experiments were carried out following standard protocols, with visualization achieved using the DAB staining method. The expression levels of the proteins were quantified by calculating the average optical density (AOD) values in the tumor sections, and the staining results were evaluated under a microscope.

### 2.15 Statistical analysis

All *in vitro* experimental data were analyzed using GraphPad Prism version 9.1. Statistical differences between groups were evaluated using the Student’s t-test. Data are expressed as *mean* ± *standard* deviation (SD). A p-value of less than 0.05 was considered statistically significant.

## 3 Results

### 3.1 Data collection

Two BLCA cohorts and corresponding clinical data were obtained from the TCGA and GEO databases. The demographic and clinical data for the training, internal testing and entire TCGA BLCA sets are summarized in **Table 1**. After filtering out the samples with missing clinical information from the TCGA BLCA dataset, a total of 407 BLCA patients, including 229 living patients and 178 patients who died at the end of follow-up, were included in this study (median follow-up time was 2.224 years). This dataset was randomly divided into a training set (n = 245, 60%) and an internal testing set (n = 162, 40%). As expected, no significant differences were found in the major clinicopathological features between the training, testing and entire TCGA BLCA datasets (**Table 1**). In addition, this study also included a GEO dataset (GSE32894) including 165 BLCA patients, which included 41.82% of deaths at the end of follow-up (median follow-up time was 4.032 years).

**Table 1:** Clinical features of the BLCA patients in the training set, testing set and validation set.

Characteristics		Training set (60%) n = 245	Testing set (40%) n = 162	All data n = 407	χ2 P value
Age	≥60	71 (28.98%)	36 (22.22%)	107 (26.29%)	0.317
	>60	174 (71.02%)	126 (77.78%)	300 (73.71%)	
Gender	female	59 (24.08%)	47 (29.01%)	106 (26.04%)	0.540
	male	186 (75.92%)	115 (70.99%)	301 (73.96%)	
Pathologic M	m0	122 (96.06%)	74 (92.50%)	196 (94.69%)	0.538
	m1	5 (3.94%)	6 (7.50%)	11 (5.31%)	
Pathologic N	N0	145 (59.18%)	92 (56.79%)	237 (58.23%)	0.891
	N1/2/3	100 (40.82%)	70 (43.21%)	170 (41.77%)	
Pathologic T	T1/2	72 (29.39%)	50 (30.86%)	122 (29.98%)	0.951
	T3/4	173 (70.61%)	112 (69.14%)	285 (70.02%)	
Stage	Stage I/II	83 (33.88%)	49 (30.25%)	132 (32.43%)	0.746
	Stage III/IV	162 (66.12%)	113 (69.75%)	275 (67.57%)	
BMI	≥24	68 (31.78%)	51 (35.66%)	119 (33.33%)	0.747
	>24	146 (68.22%)	92 (64.34%)	238 (66.67%)	
Cigarettes per day	≤2	75 (55.15%)	51 (60.00%)	126 (57.01%)	0.778
	>2	61 (44.85%)	34 (40.00%)	95 (42.99%)	
Time	≤2	165 (67.35%)	102 (62.96%)	267 (65.60%)	0.660
	>2	80 (32.65%)	60 (37.04%)	140 (34.40%)	
Status	0	136 (55.51%)	93 (57.41%)	229 (56.27%)	0.931
	1	109 (44.49%)	69 (42.59%)	178 (43.73%)	

### 3.2 Construction and validation of the prognostic model according to the CSRGs in BLCA patients

**Figure 1:**
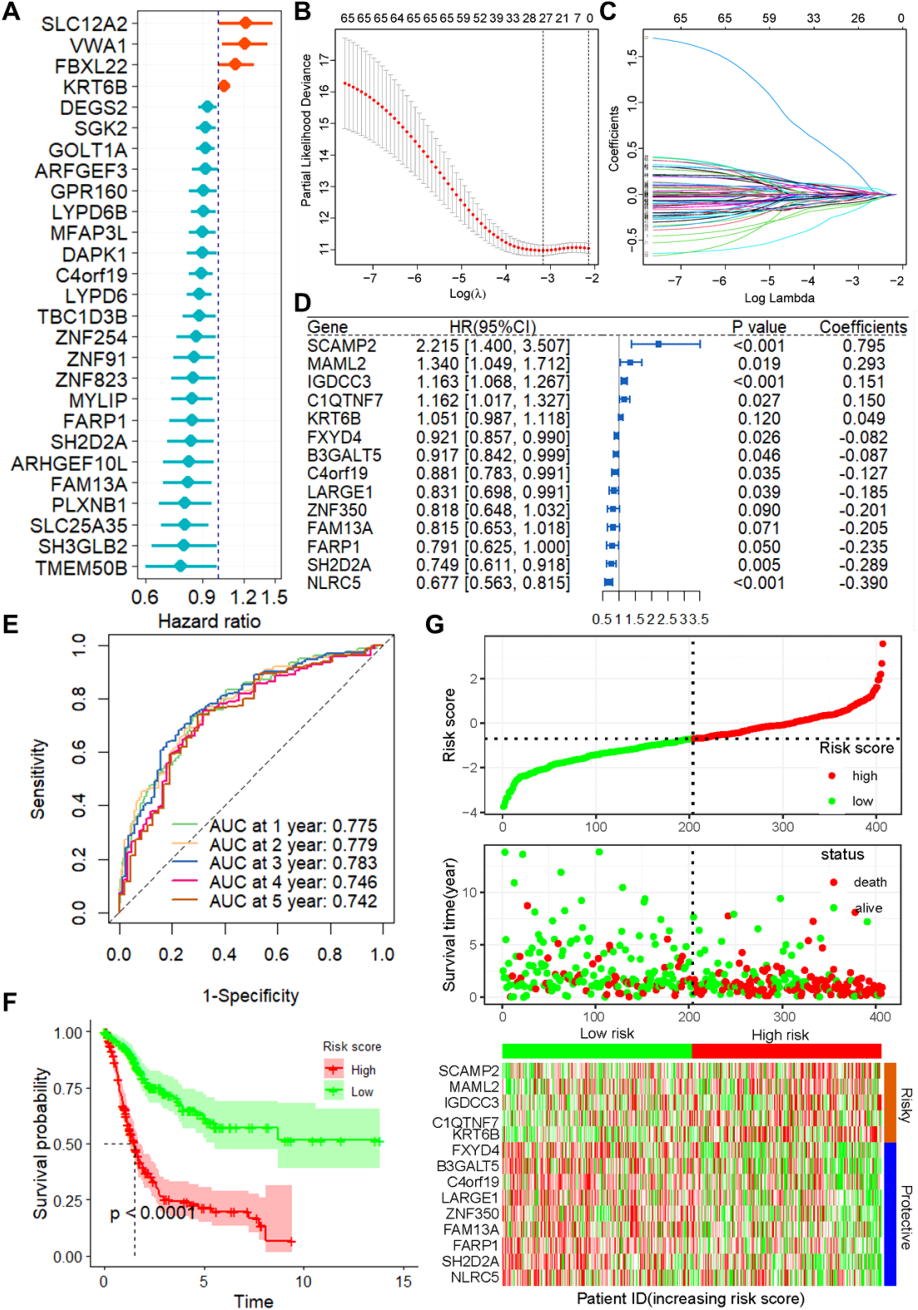
Construction of the prognostic model of CSRGs. **(A)** Univariate Cox regression analysis for the selection of CSRGs correlated with the OS of BLCA patients. **(B)** Partial likelihood deviance of OS for the LASSO coefficient profiles. **(C)** LASSO coefficient profiles of the 23 DRGs for OS. **(D)** Forest plot showing the multivariate Cox regression analysis of 14 CSRGs. **(E)** ROC curves for 1-year OS in the training set. **(F)** K–M curve of OS in the training group. **(G)** Risk score distribution and survival status of the training group, and the heatmap showing the expression of 14 CSRGs in the training group. Genes with HR values > 1 are considered “Risky,” while those< 1 are deemed “Protective”. CSRGs, cisplatin sensitivity-related genes; OS, Overall survival; ROC, receiver operating characteristic curve; BLCA, bladder cancer.

Correlation analysis revealed 381 positively and 735 negatively correlated CSRGs, meeting the criteria of an absolute correlation coefficient greater than 0.3 and a P-value below 0.05 (**Supplementary Figure S1**). Using univariate Cox regression analysis on the TCGA training set, 27 prognosis-related CSRGs were identified (**Figure 1A**). Subsequent LASSO-penalized Cox analysis narrowed these down to 14 CSRGs suitable for multivariate analysis (**Figures 1B, C**). A stepwise multivariate Cox proportional hazard model was constructed using the likelihood-ratio forward selection method, achieving the highest level of statistical significance. From these, a prognostic risk model for BLCA patients was developed based on the expression levels of the 14 CSRGs: risk score = (-0.289 × SH2D2A exp) + (-0.185 × LARGE1 exp) + (-0.205 × FAM13A exp) + (0.795 × SCAMP2 exp) + (-0.390 × NLRC5 exp) + (-0.082 × FXYD4 exp) + (-0.235 × FARP1 exp) + (-0.127 × C4orf19 exp) + (0.150 × C1QTNF7 exp) + (0.151 × IGDCC3 exp) + (-0.087 × B3GALT5 exp) + (0.293 × MAML2 exp) + (0.049 × KRT6B exp) + (-0.201 × ZNF350 exp) (**Figure 1D**). ROC analysis confirmed that this risk score significantly predicts overall survival (OS) in BLCA patients, with AUCs exceeding 0.775 at 1, 2, and 3 years (**Figure 1E**). Based on the median risk score, patients in the training set were classified into low- and high-risk categories. Kaplan-Meier survival analysis demonstrated that the low-risk group had a significantly better OS (**Figure 1F**). **Figure 1G** show the distribution of risk scores, survival status, and survival times across the two risk categories, along with the relative expression of the 14 CSRGs in each patient.

**Figure 2:**
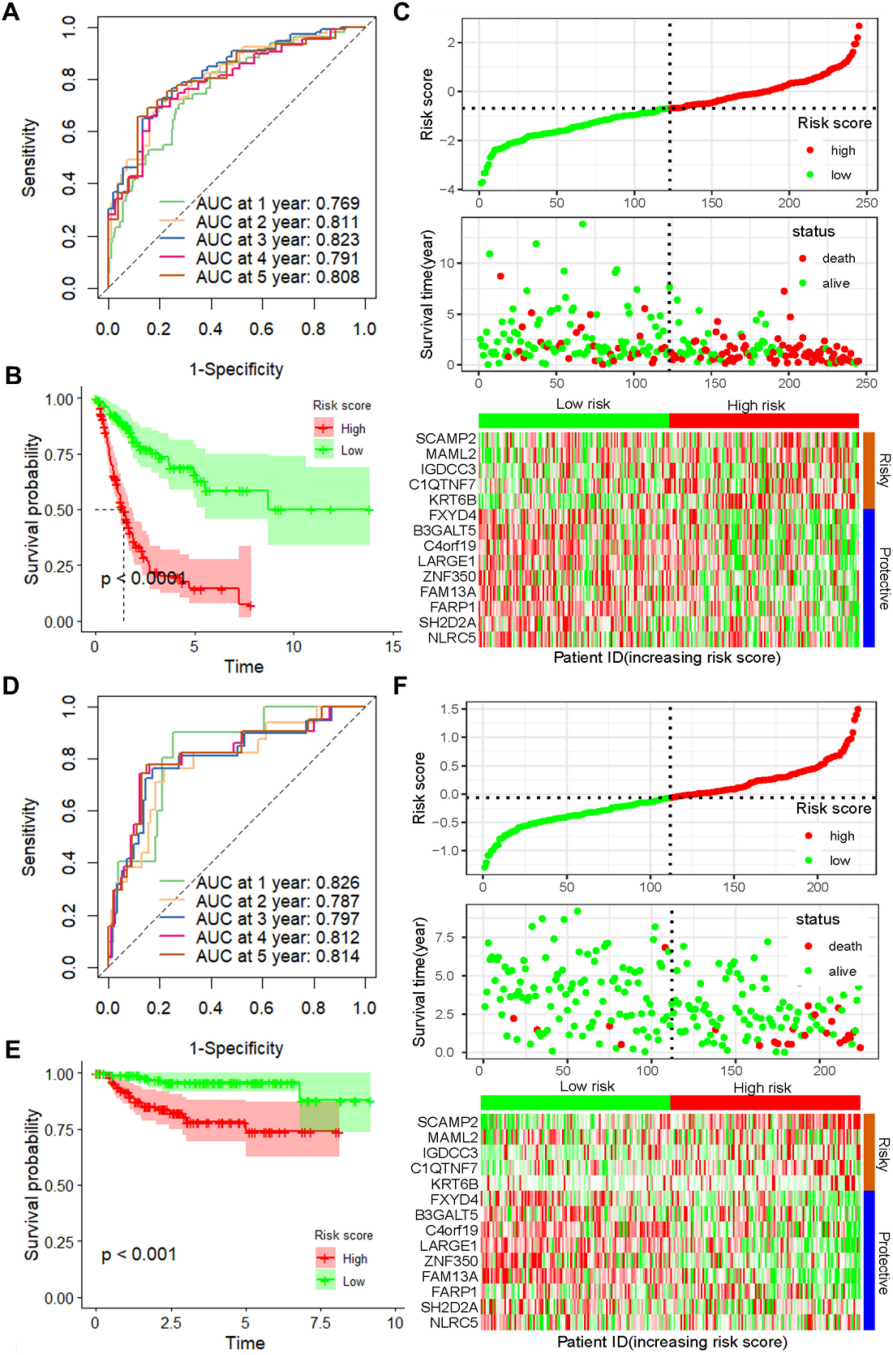
Validation of the prognostic model with 14 CSRGs constructed from the training dataset. **(A)** ROC curves for OS in the entire TCGA-BLCA dataset. **(B)** K–M curves of OS in the entire TCGA-BLCA dataset. **(C)** Risk score distribution, survival status and expression of 14 CSRGs in the entire TCGA-BLCA dataset. **(D)** ROC curves for OS in GSE32894 dataset. **(E)** K–M curves of OS in GSE32894 dataset. **(F)** Risk score distribution, survival status and expression of 14 CSRGs in GSE32894 dataset. CSRGs, cisplatin sensitivity-related genes; ROC, dependent receiver operating curve; TCGA, the cancer genome map; BLCA, bladder cancer; OS, overall survival.

To further verify the accuracy and reliability of the prognostic model obtained from the training set, we applied it to the internal testing set and other independent validation cohorts, *viz*. GSE32894. ROC curves and K-M curve indicated that the risk score was an effective predictor of the OS of BLCA patients in the internal testing set (n = 162, **Supplementary Figure S2**). In addition, the same observation was also found in the entire TCGA BLCA dataset (**Figures 2A–C**), as well as in the GSE32894 validation cohort (**Figures 2D–F**).

### 3.3 The DRG risk score is independent of clinical features

**Figure 3:**
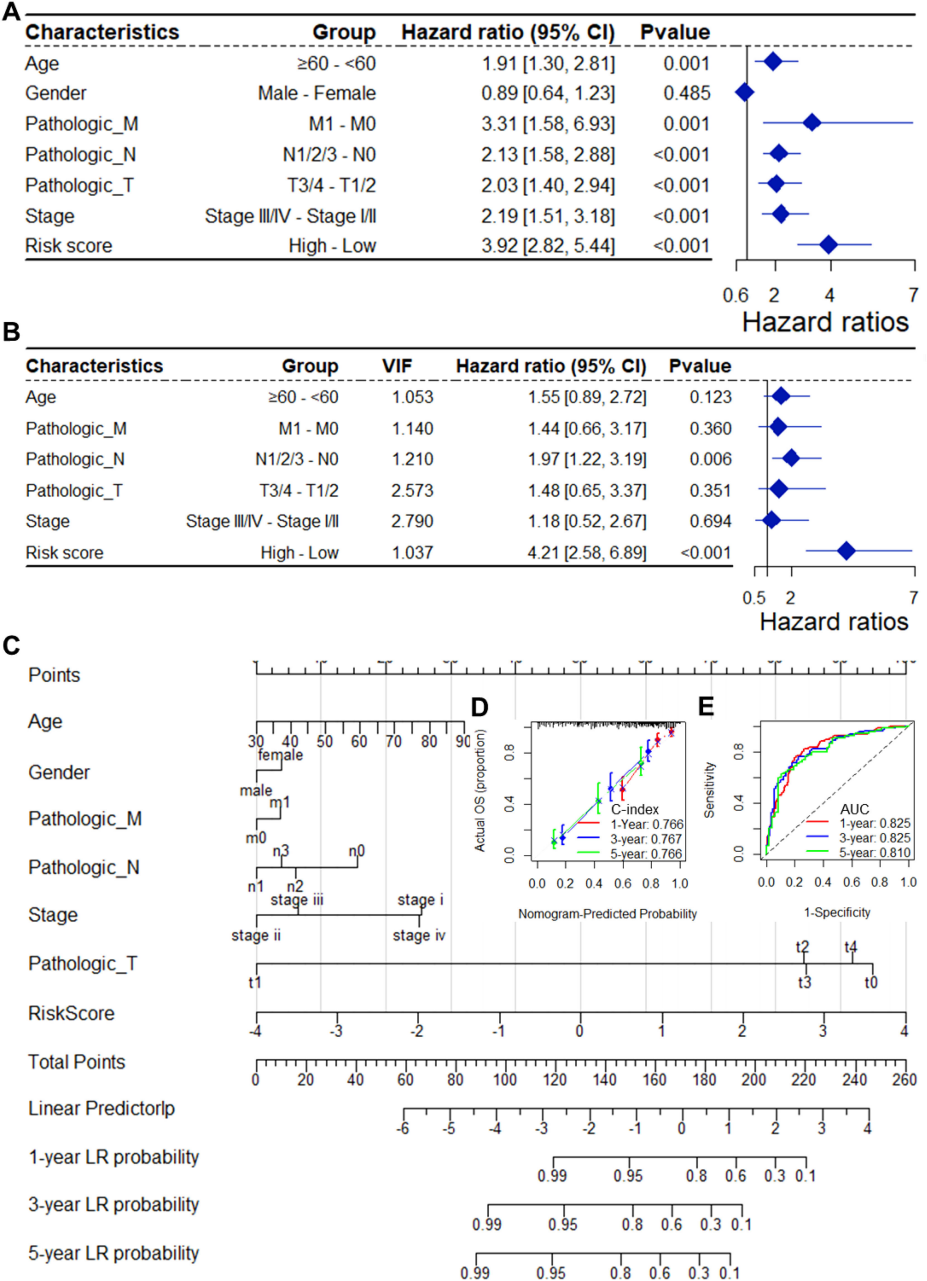
The CSRGs risk score was an independent prognostic factor for OS in the TCGA-BLCA dataset. Univariate **(A)** and multivariate **(B)** Cox regression analyses of the risk score and clinicopathological features for overall survival in the TCGA-BLCA dataset. **(C)** The nomogram consists of the 14-gene risk score and 6 clinical indicators based on the TCGA-BLCA dataset. The points from these variables are combined, and the locations of the total points are determined. The total points projected on the bottom scales indicate the probabilities of 1-year, 3-year and 5-year overall survival. Calibration plots **(D)** and receiver operating characteristic (ROC) curves **(E)** were used to validate the prognostic nomogram constructed based on the TCGA- BLCA dataset. CSRGs, cisplatin sensitivity-related genes; ROC, dependent receiver operating curve; TCGA, the cancer genome map; BLCA, bladder cancer; OS, overall survival.

As depicted in **Supplementary Table S1**, the CSRGs risk score was related to pathologic T and tumor stage in the TCGA-BLCA dataset. To assess whether the risk score is an independent indicator in BLCA patients, the effect of each clinicopathologic feature on OS was analyzed by univariate Cox regression (**Figure 3A**). As shown in **Figure 3B**, after collinearity test and multivariable adjustment, the risk score remained a powerful and independent factor in the entire TCGA-BLCA dataset. Moreover, the risk score was verified as an independent factor based on the GSE32894 dataset (**Supplementary Figures S3A, B**). According to the subgroups classified by pathologic T stage, the OS of the low-risk group was superior to that of the high-risk group (**Supplementary Figure S4**).

To ensure the robustness and practicability of the 14-CSRGs prognostic model, a prognostic nomogram for predicting overall survival in BLCA patients was established using the TCGA-BLCA and GSE32894 datasets (**Figure 3C**, **Supplementary Figure S3C**). Major clinicopathological features and risk scores were included in the nomogram. The nomogram was internally validated by computing the bootstrap C-index (≥ 0.766 in TCGA-BLCA and ≥ 0.923 in GSE32894) and a calibration plot (**Figure 3D**, **Supplementary Figure S3D**). The ROC curve confirmed that the score calculated based on the nomogram was highly predictive of overall survival, with AUCs of 0.825 and 0.917 at 1 year in the TCGA-BLCA and GSE32894 cohorts, respectively (**Figure 3E**, **Supplementary Figure S3E**).

### 3.4 Risk score is associated with tumor progression

**Figure 4:**
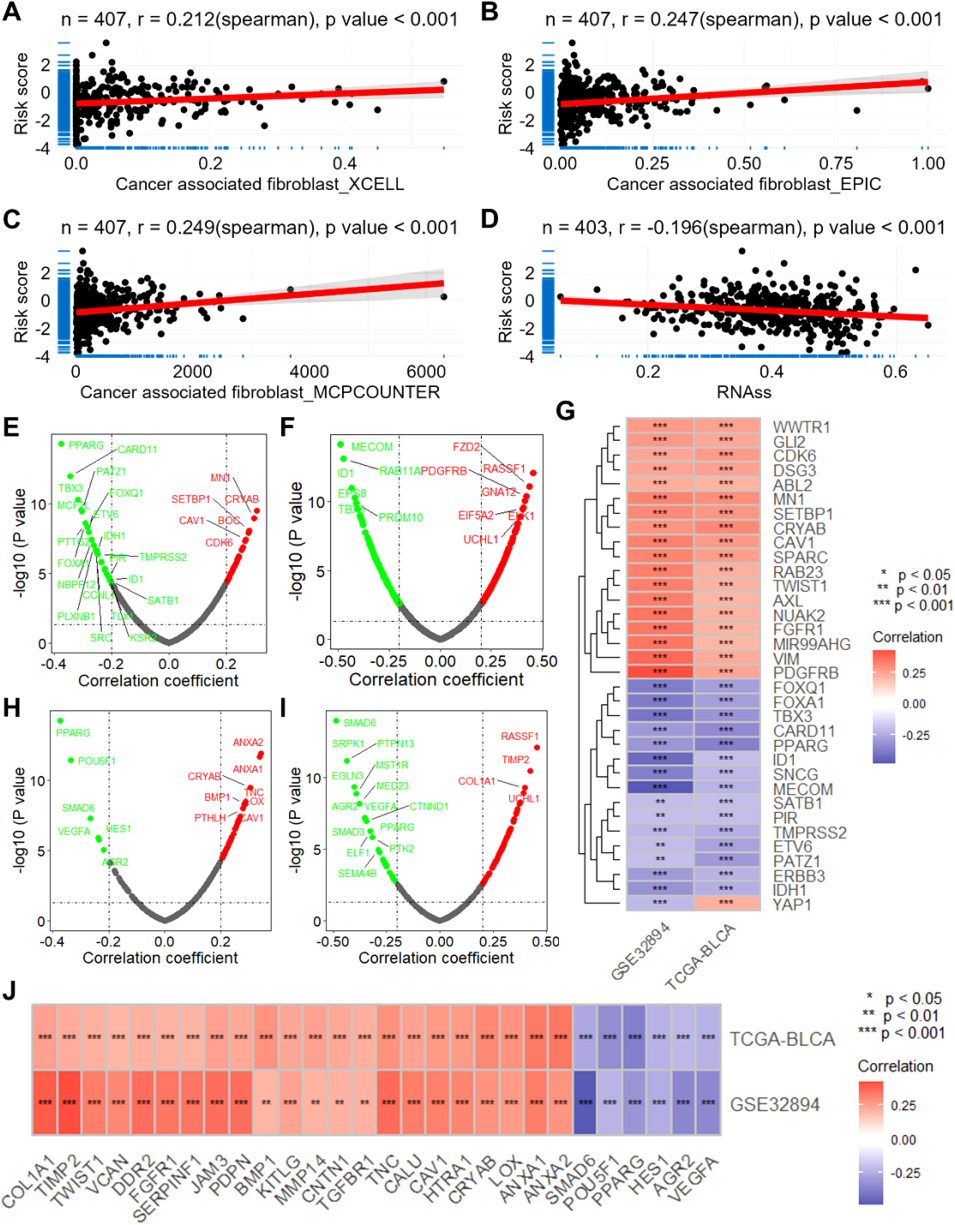
The CSRGs risk score is associated with the infiltration of macrophage and cancer associated fibroblast and the expression of oncogenes and metastasis-associated genes. Scatter plots show the correlations between CSRGs risk score and the infiltration score of cancer associated fibroblast calculated by XCELL **(A)**, EPIC **(B)** and MCPCOUNTER **(C)** algorithms. **(D)** Scatter plot show the correlation between CSRGs risk score and tumor stemness score. Volcano plots show the correlation analysis between CSRGs risks score and oncogenes in TCGA-BLCA **(E)** and GSE32894 **(F)** datasets. **(G)** A heatmap illustrates the intersection of oncogenes associated with risk scores in the two datasets. **(D)** Scatter plot show the correlation between CSRGs risk score and tumor stemness score. Volcano plots show the correlation analysis between CSRGs risks score and metastasis associated genes in TCGA-BLCA **(H)** and GSE32894 **(I)** datasets. **(J)** A heatmap illustrates the intersection of metastasis associated genes associated with risk scores in the two datasets. CSRGs, cisplatin sensitivity-related genes; TCGA, the cancer genome map; BLCA, bladder cancer.

To assess the biological significance of CSRGs risk scores, we conducted a series of correlation analyses. Firstly, utilizing the XCELL, EPIC, and MCPCOUNTER algorithms, we computed the infiltration scores of tumor-associated fibroblasts for each sample in the TCGA-BLCA dataset. Subsequent correlation analysis revealed a significant positive correlation between CSRGs risk scores and these scores (all p<0.001, r>0.2, **Figures 4A–C**). Moreover, the risk scores exhibited a significant negative correlation with tumor stemness scores calculated using the RNAss algorithm (all p<0.001, r = -0.196, **Figure 4D**). Furthermore, we observed a significant correlation between risk scores and the expression of multiple oncogenes (**Figures 4E–G**) as well as genes associated with tumor metastasis (**Figures 4H–J**).

### 3.5 Risk score links to biological functions and pathways

**Figure 5:**
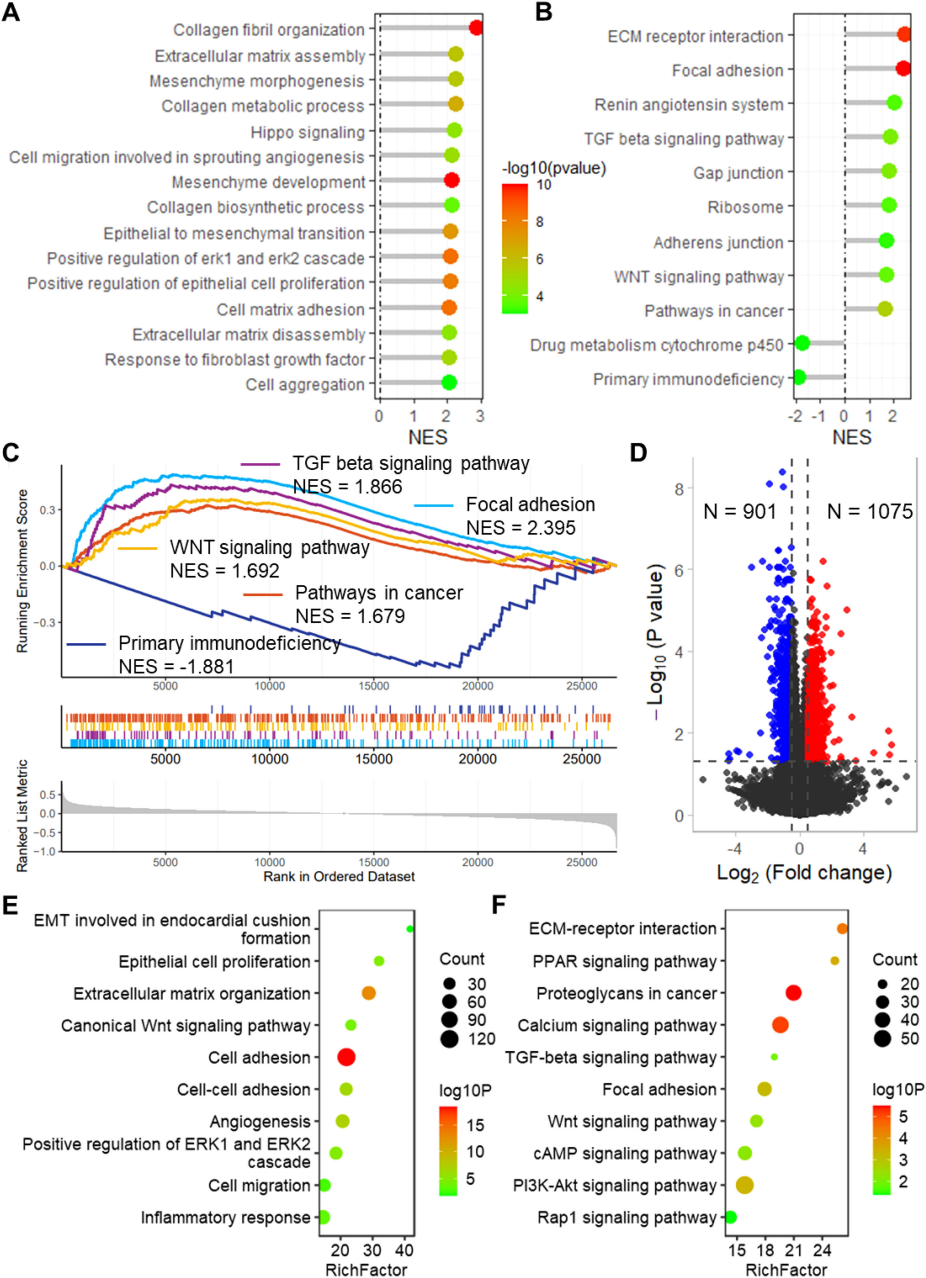
Enrichment analysis revealed that the risk score links to biological functions and pathways. Lollipop plots show the GSEA enrichment analysis of risk score for biological processes **(A)** and KEGG pathways **(B)**. **(C)** GSEA plots show the GSEA results of TGF beta signaling pathway, focal adhesion, WNT signaling pathway, pathways in cancer and primary immunodeficiency. **(D)** Volcano plot shows the differentially expressed genes between high and low risk groups in TCGA-BLCA dataset. Lollipop plots show the enrichment analysis of the differentially expressed genes for biological processes **(E)** and KEGG pathways **(F)**. TCGA, the cancer genome map. BLCA, bladder cancer; GSEA, Gene Set Enrichment Analysis; KEGG, Kyoto Encyclopedia of Genes and Genomes.

To evaluate the biological significance of CSRGs risk score in BLCA, enrichment analysis was performed. The GO enrichment analysis revealed that the risk score is related to several vital biological processes, including extracellular matrix assembly, mesenchyme morphogenesis, collagen metabolic process, epithelial to mesenchymal, positive regulation of epithelial cell proliferation and cell matrix adhesion (**Figure 5A**). Also, the CSRGs risk score is significantly related to many cancer-associated pathways (**Figures 5B, C**), including ECM receptor interaction, focal adhesion (NES = 2.395), TGF-β signaling pathway (NES = 1.966), WNT signaling pathway (NES = 1.792), pathway in cancer (NES = 1.779), and primary immunodeficiency (NES = -1.881). Moreover, a total of 901 down-regulated and 1075 upregulated genes were identified between high and low risk groups (**Figure 5D**). The enrichment analysis revealed that these genes enriched in many important biological processes and KEGG pathways, including cell proliferation, cell adhesion, cell migration, as well as TGF-β, WNT, cAMP, PI3K-Akt and Rap1 signaling pathways (**Figures 5E, F**). In addition, the risk score was also found to be associated with multiple cancer-related biological processes and KEGG pathways in GSE32894 (**Supplementary Figure S5**).

### 3.6 SCAMP2 is a key gene in the model with vital biological functions

**Figure 6:**
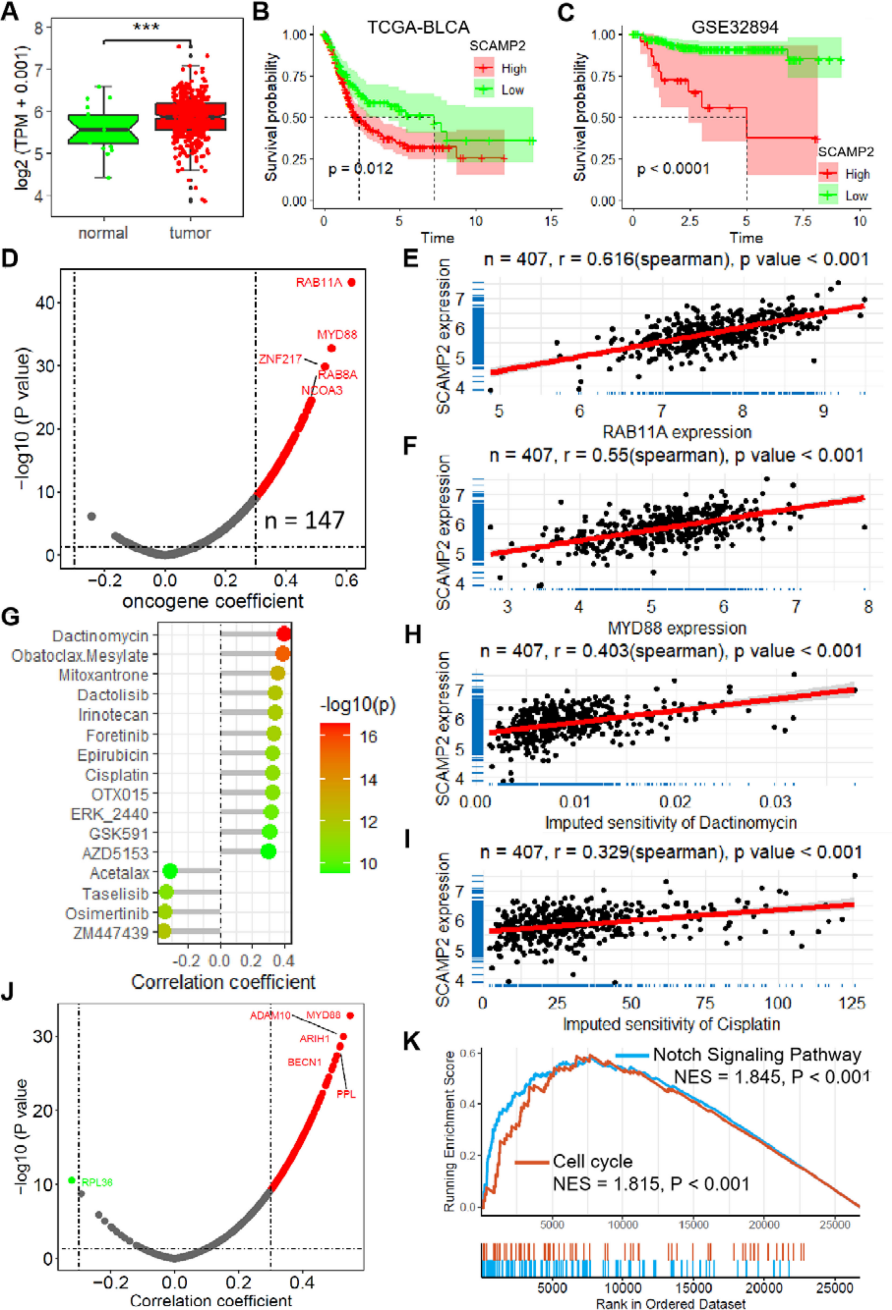
SCAMP2 is a key gene in the model with vital biological functions. **(A)** Box plot shows the expression of SCAMP2 in TCGA-BLCA dataset. **(B)** KM plots of the samples with low and high expression of SCAMP2 in TCGA-BLCA **(B)** and GSE32894 **(C)** datasets. **(D)** Scatter plot shows the correlation between the expression of SCAMP2 and oncogenes in TCGA-BLCA dataset. Scatter plots show the correlation between the expression of SCAMP2 and RAB11A **(E)** and MYD88 **(F)**. **(G)** Lollipop plot shows the correlation between the expression of SCAMP2 and the sensitivity of anti-tumor drugs in TCGA-BLCA dataset. Scatter plots show the correlation between the expression of SCAMP2 and the sensitivity of dactinomycin **(H)** and cisplatin **(I)**. **(J)** Scatter plot shows the correlation between the expression of SCAMP2 and cisplatin resistance related genes in Platinum database. **(K)** GSEA plots show the enrichment results of Notch Signaling Pathway and Cell cycle in TCGA-BLCA dataset; TCGA, the cancer genome map. BLCA, bladder cancer; GSEA, Gene Set Enrichment Analysis.

The expression of SCAMP2, the member with the largest coefficient in the risk model, was significantly positively correlated with the risk score (**Supplementary Figure S6**). In the TCGA-BLCA dataset, SCAMP2 expression in tumor tissues was significantly higher than in normal tissues (**Figure 6A**), and low expression of SCAMP2 in samples from both the TCGA-BLCA and GSE32894 datasets was associated with significantly better prognosis compared to high expression samples (**Figures 6B, C**). Further correlation analysis revealed that SCAMP2 expression was significantly positively correlated with the expression of numerous oncogenes (r > 0.3, **Figure 6D**), with the two most correlated genes being RAB11A (r = 0.616, **Figure 6E**) and MYD88 (r = 0.550, **Figure 6F**). Additionally, we further analyzed the correlation between SCAMP2 expression and the sensitivity to 198 anticancer drugs, revealing significant correlations with multiple anticancer drugs (**Figure 6G**), including dactinomycin (r = 0.403, **Figure 6H**) and cisplatin (r = 0.329, **Figure 6I**). Furthermore, based on the Platinum database, we obtained genes associated with cisplatin resistance, and further correlation analysis demonstrated that SCAMP2 expression was significantly positively correlated with multiple resistance genes (r > 0.3, **Figure 6J**). Functionally, GSEA analysis revealed that SCAMP2 was associated with multiple tumor-related signaling pathways, including the Notch Signaling Pathway and Cell cycle (**Figure 6K**).

### 3.7 SCAMP2 contributes to proliferation, migration and cisplatin sensitivity in bladder cancer

**Figure 7:**
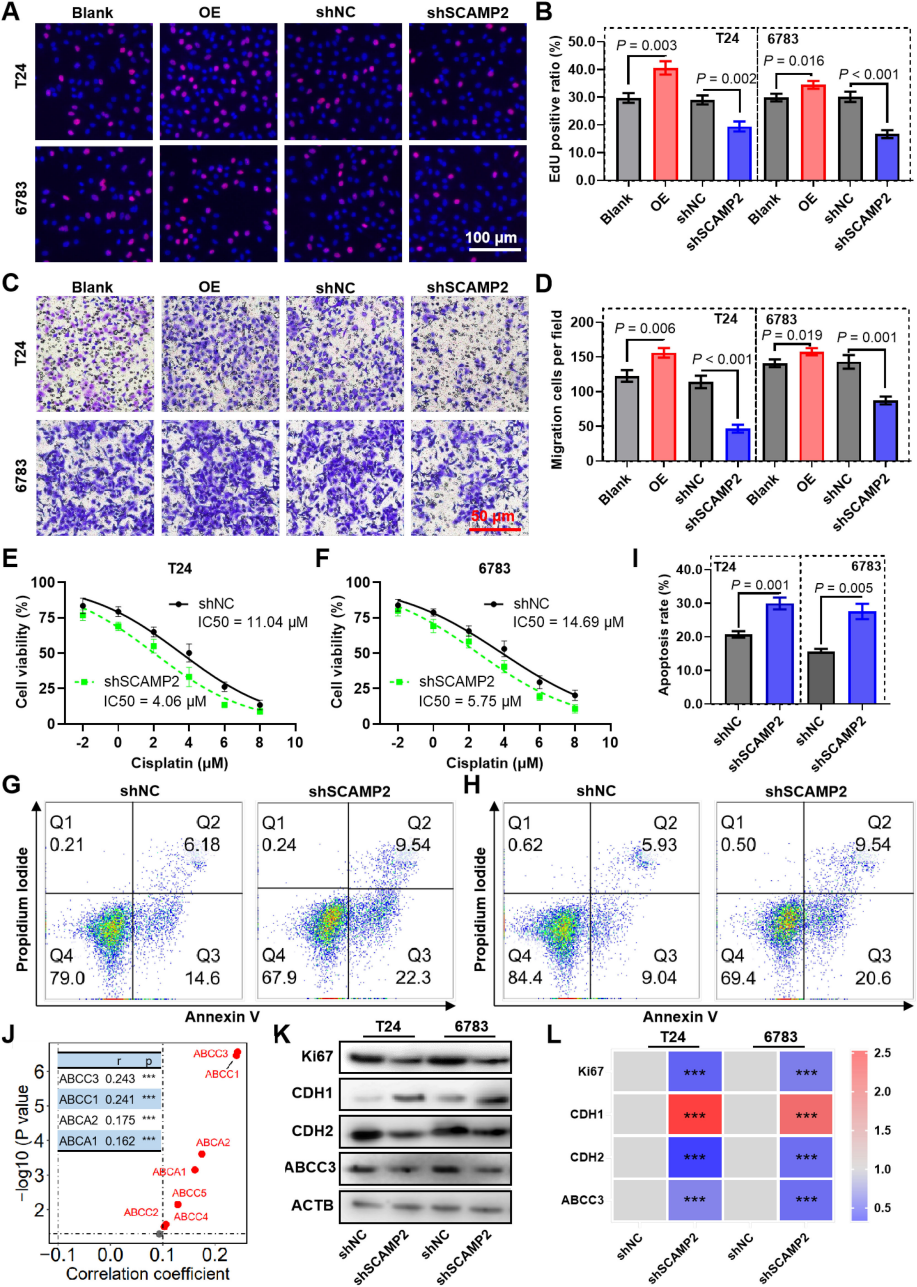
SCAMP2 contributes to proliferation, migration and cisplatin sensitivity in bladder cancer. Representative images **(A)** and the quantified result **(B)** of the EdU cell proliferation assay for bladder cancer cells knockdown or expressed SCAMP2. Representative images **(C)** and the quantified result **(D)** of the transwell cell migration assay for bladder cancer cells knockdown or expressed SCAMP2. **(E-F)** CCK-8 assay reveals the cell viability of SCAMP2 knockdown cells induced by 2 μM cisplatin treatment. Representative images **(G-H)** and the quantified result **(I)** of the apoptosis assay for SCAMP2 knockdown cells induced by 2 μM cisplatin treatment. **(J)** The scatter plot illustrates the correlation between SCAMP2 expression and ABC transporter expression in the BLCA dataset from the TCGA database. Typical Western blot images show the changes in Ki67, CDH1, and CDH2 expression after SCAMP2 knockdown in bladder cancer cells **(K)**, along with the corresponding statistical results (L). *** P< 0.001.

To evaluate the biological function of SCAMP2 in BLCA cells, we constructed shRNA plasmids to knock it down, as well as a plasmid to overexpress it (**Supplementary Figure S7**). The EdU assay demonstrated that SCAMP2 knockdown could significantly inhibit the proliferation of bladder cancer cells (**Figures 7A, B**). Conversely, overexpressed SCAMP2 significantly promotes the proliferation ability (**Figures 7A, B**). When considering cell migration, the transwell migration assay indicated that knockdown of SCAMP2 significantly reduced the migrated cells, while overexpression significantly increased it (**Figures 7C, D**). Also, the results of CCK-8 assay revealed that SCAMP2 knockdown significantly promoted the sensitivity of cisplatin (**Figures 7E, F**). The further cell apoptosis assay revealed that SCAMP2 knockdown significantly increased the cell apoptosis induced the treatment of 2 μM cisplatin in T24 and 6783 bladder cancer cells (**Figures 7G–I**). The correlation analysis results indicate that SCAMP2 expression is significantly positively correlated with the expression of several ABC transporters (r > 0.1, P< 0.05, **Figure 7J**), with the highest correlations observed for ABCC3 (r = 0.243) and ABCC1 (r = 0.241). Western blotting results demonstrate that, following SCAMP2 knockdown in bladder cancer cells, the proliferation marker Ki67 is significantly downregulated, the epithelial marker CDH1 is significantly upregulated, the mesenchymal marker CDH2 is significantly downregulated, and the drug resistance gene ABCC3 is significantly downregulated (**Figures 7K, L**).

### 3.8 SCAMP2 regulates cisplatin resistance in bladder cancer via the NOTCH signaling pathway

**Figure 8:**
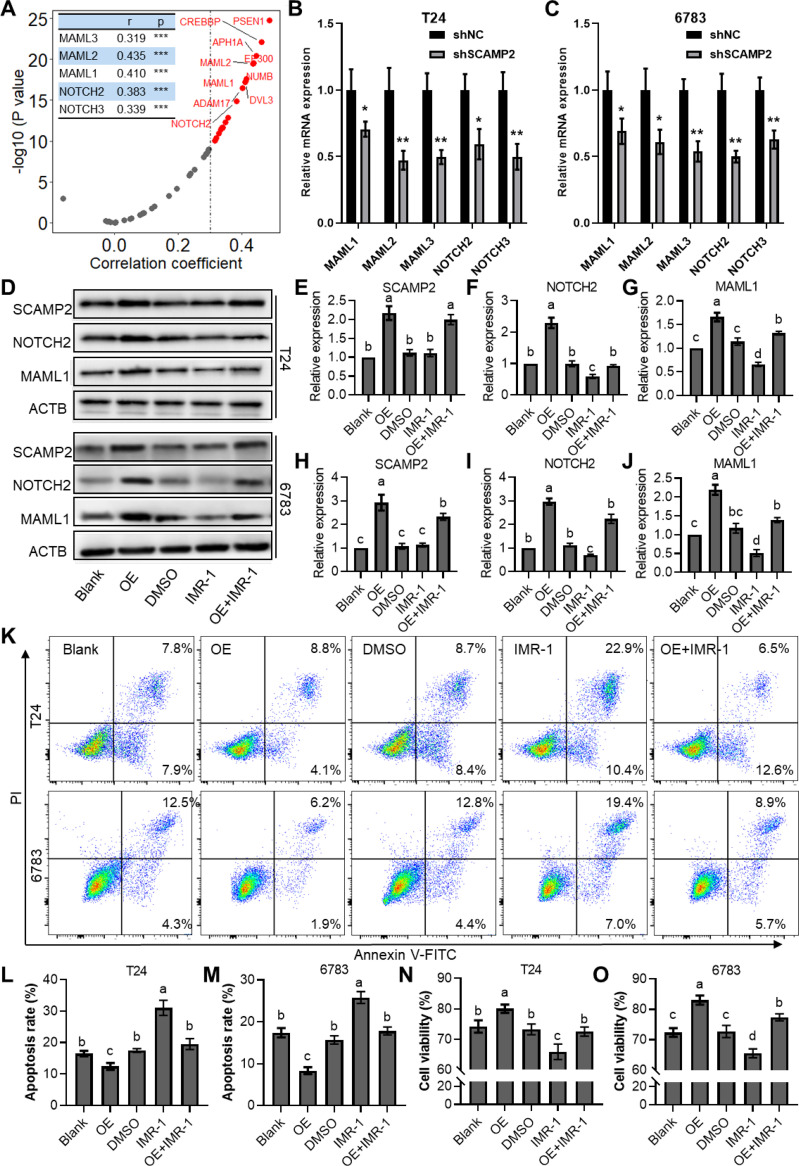
SCAMP2 regulates cisplatin resistance in bladder cancer via the notch signaling pathway. **(A)** Scatter plots showing the correlation between SCAMP2 and genes in the NOTCH signaling pathway in the TCGA-BLCA dataset; qPCR results indicating changes in the expression of MAML1/2/3 and NOTCH2/3 after SCAMP2 knockdown in T24 **(B)** and 6783 **(C)** cells; Representative images **(D)** and quantitative data **(E)** of Western blot analysis showing changes in the expression of SCAMP2, NOTCH2, and MAML1 after 1 μM cisplatin treatment, SCAMP2 overexpression, and/or IMR-1 addition; Flow cytometry images **(K)** and statistical analysis (L-M) of apoptosis rates in each group; (N-O) CCK-8 assay results showing changes in cell viability in each group. TCGA, the cancer genome map; BLCA, bladder cancer.

Correlation analysis revealed that SCAMP2 is significantly positively correlated with several genes in the NOTCH signaling pathway (**Figure 8A**), including MAML1 (r = 0.410), MAML2 (r = 0.435), MAML3 (r = 0.319), NOTCH2 (r = 0.383), and NOTCH3 (r = 0.339). Further qPCR results indicated that knockdown of SCAMP2 in two bladder cancer cell lines significantly reduced the expression of these genes (**Figures 8B, C**). Additionally, we overexpressed SCAMP2 and/or added the NOTCH pathway inhibitor IMR-1 in bladder cancer cells treated with 1 μM cisplatin. Western blot results demonstrated that overexpression of SCAMP2 significantly upregulated NOTCH2 and MAML1, while addition of IMR-1 significantly downregulated these genes. IMR-1 treatment also rescued the upregulation of NOTCH2 and MAML1 induced by SCAMP2 overexpression (**Figures 8D–J**). At the cellular level, we assessed apoptosis and cell viability to verify this regulatory mechanism. The results showed that SCAMP2 overexpression significantly reduced apoptosis (**Figures 8K–M**) and enhanced cell viability (**Figures 8N, O**). Conversely, IMR-1 treatment significantly increased apoptosis and decreased cell viability, rescuing the reduced apoptosis (**Figures 8K–M**) and increased viability (**Figures 8N, O**) induced by SCAMP2 overexpression.

### 3.9 SCAMP2 overexpression enhances cisplatin sensitivity in bladder cancer *in vivo*

**Figure 9:**
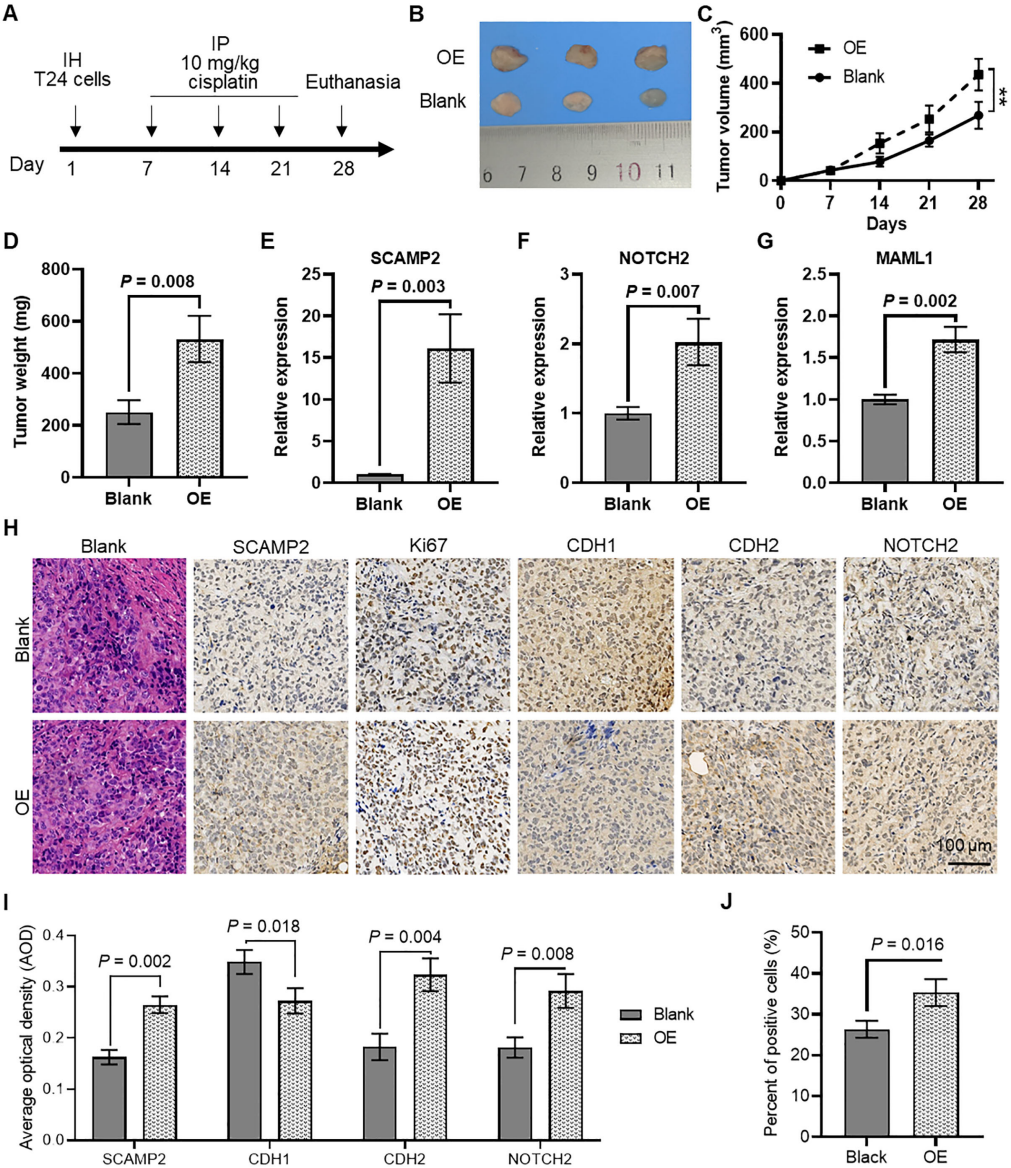
*In vivo* evidence that SCAMP2 overexpression regulates cisplatin sensitivity in bladder cancer tissues. **(A)** Timeline of *in vivo* experiments; **(B)** Images of subcutaneous tumors formed in nude mice using T24 cells with stable SCAMP2 overexpression; **(C)** Growth curve of subcutaneous tumors in nude mice; **(D)** Differences in tumor weight between the two groups of mice at the end of the experiment; **(E)** qPCR analysis of SCAMP2 **(E)**, NOTCH2 **(F)**, and MAML **(G)** in tumors from the two groups of mice; **(H)** Immunohistochemical staining analysis of SCAMP2, Ki67, CDH1, CDH2, and NOTCH2 in tumor tissues from the two groups of mice; **(I)** AOD statistical results of immunohistochemical images for SCAMP2, CDH1, CDH2, and NOTCH2; **(J)** Statistical results of the proportion of Ki67-positive cells in immunohistochemical images. IH, intraperitoneal injection; IP, intraperitoneal injection; AOD, average optical density.

The further *in vivo* experiments were conducted to evaluate the regulatory effect of SCAMP2 overexpression on cisplatin sensitivity in bladder cancer tissues (**Figure 9A**). As shown in **Figure 9B**, subcutaneous tumors formed in nude mice using T24 cells with stable SCAMP2 overexpression demonstrated significant tumor formation. The tumor growth curve (**Figure 9C**) indicated that following treatment with 10 mg/mL cisplatin, the tumor growth rate in the SCAMP2 overexpression group was significantly higher than that in the control group. At the end of the experiment, the tumor weight in the SCAMP2 overexpression group was significantly higher than that in the control group (**Figure 9D**). qPCR analysis revealed that SCAMP2 overexpression significantly increased the expression of NOTCH2 and MAML (**Figures 9E–G**). Furthermore, immunohistochemical staining analysis showed that the average optical density (AOD) of SCAMP2, CDH2, and NOTCH2 in the SCAMP2 overexpression group was significantly higher than that in the control group, while CDH1 was significantly decreased (**Figures 9H, I**). Additionally, statistical analysis of the proportion of Ki67-positive cells (**Figure 9I**) indicated a significant increase in the proportion of Ki67-positive cells in the SCAMP2 overexpression group (**Figure 9J**), further supporting the critical role of SCAMP2 in regulating cisplatin sensitivity in bladder cancer tissues.

## 4 Discussion

Bladder cancer, as a common malignant tumor of the urinary system, has long been a focus of concern regarding patient prognosis. Despite advancements in treatment modalities, platinum resistance remains a significant obstacle to achieving favorable outcomes for bladder cancer patients (20). Therefore, studies focusing on constructing prognostic models based on platinum resistance-related genes hold crucial clinical significance. Previous research has proposed various prognostic models for bladder cancer based on different molecular features. Among these, prognostic models based on muscle-invasive related genes (MIRDGs) (21), m^6^A-immune-related long non-coding RNA (lncRNA) signatures (22), and epithelial-mesenchymal transition (EMT)-related gene signatures (23) are notable. While these models demonstrate certain predictive performance in the TCGA-BLCA dataset, their AUC values are relatively modest (AUC = 0.753, 0.743 and 0.659 for 1-year OS, separately), limiting their clinical utility.

In our study, we developed a novel prognostic model based on cisplatin sensitivity related genes (CSRGs). This model exhibited excellent predictive performance in the TCGA-BLCA dataset, with a 3-year overall survival (OS) AUC of 0.823. Furthermore, in an independent external validation set, the model demonstrated high predictive performance, with a 3-year AUC of 0.797. Additionally, when other independent prognostic factors were integrated to construct a nomogram model, which showed superior predictive ability. In both datasets, the nomogram model yielded 3-year OS AUCs of 0.810 and 0.946, respectively. Nomograms are commonly employed in cancer prognosis due to their capacity to condense complex statistical predictive models into a singular numerical estimate reflecting the likelihood of an event, such as death or recurrence, personalized to an individual patient’s characteristics (24). This CSRGs prognostic model not only demonstrates significant predictive performance in clinical prognosis assessment but also reveals certain biological significance. Firstly, a close association was observed between the model risk score and infiltration of Cancer-Associated Fibroblasts (CAFs), which plays crucial roles in the tumor microenvironment, exerting pivotal effects on tumor initiation and progression (25, 26). Furthermore, we found a significant correlation between the model risk score and the expression of oncogenes and metastasis-related genes. Aberrant expression of oncogenes is closely associated with tumor cell proliferation and metastasis, while the expression of metastasis-related genes is closely related to distant metastasis and prognosis of tumors (27).

Our study reveals that SCAMP2, as a gene with the highest coefficient in our model, exhibits elevated expression in bladder cancer and holds significant biological significance. Additionally, we found a significant correlation between the expression of SCAMP2 and the sensitivity to various anti-cancer drugs, including cisplatin. The further *in vivo* and *in vitro* experiments also confirmed the regulatory role of SCAMP2 in bladder cancer drug resistance and proliferation. Although reports on SCAMP2 are currently limited, the only study suggesting SCAMP2/5 as diagnostic and prognostic markers for acute myeloid leukemia (28). Furthermore, members of the SCAMP family are gaining attention, with dysregulation observed in various human malignancies, such as hepatocellular carcinoma, suggesting their potential importance in tumorigenesis and progression (29–31). NOTCH signaling is deeply involved in the development and homeostasis of various tissues and organs, and its aberrations can lead to both cancerous and non-cancerous diseases. In the context of cancer, NOTCH signaling can both promote and inhibit tumor progression in various types of cancer. Additionally, NOTCH mutations have been proposed as predictive biomarkers for immune checkpoint blockade therapies in several cancers (32, 33). Furthermore, the epithelial-mesenchymal transition (EMT) process, which converts quiescent epithelial cells into motile mesenchymal cells and alters intercellular adhesion as well as the extracellular matrix, significantly promotes chemotherapy resistance by facilitating tumor cell invasion (34). Previous studies have indicated that dysregulation of Notch signaling plays a crucial role in EMT and tumor aggressiveness (35). In our study, SCAMP2 overexpression was found to promote the EMT process, evidenced by the downregulation of the epithelial marker CDH1 and the upregulation of the mesenchymal marker CDH2, thereby enhancing cisplatin resistance in bladder cancer.

Our novel prognostic model based on cellular senescence-related genes (CSRGs) demonstrates excellent predictive performance in bladder cancer. Also, we identified SCAMP2 as a key gene, which regulates cisplatin resistance through the NOTCH signaling pathway. Our research provides a promising framework for improving bladder cancer prognosis and advancing personalized therapeutic interventions.

## Data Availability

The original contributions presented in the study are included in the article/**Supplementary Material**, further inquiries can be directed to the corresponding author/s.
