# Supplementary material for: Identification of SCAMP2 as a regulator of NOTCH signaling in cisplatin resistance through a novel prognostic model for bladder cancer
Source: Front Immunol. 2025 May 8;16:1573412. doi: 10.3389/fimmu.2025.1573412 (PMC12095277; doi:10.3389/fimmu.2025.1573412)
Supplement: Supplementary file 1 [file DataSheet1.docx]

Supplementary Material

## Supplementary Figures


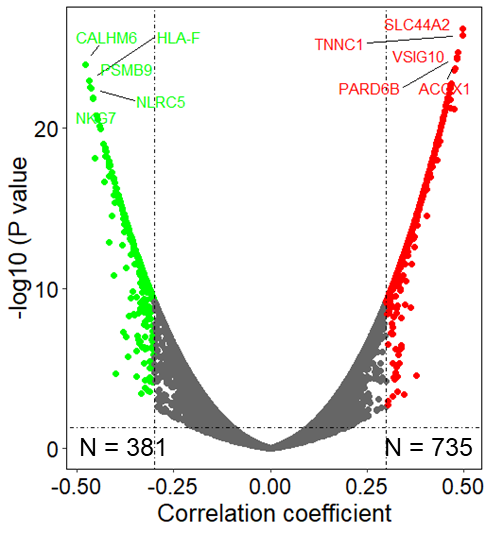


**Figure S1.** Identify cisplatin resistance related genes.

**
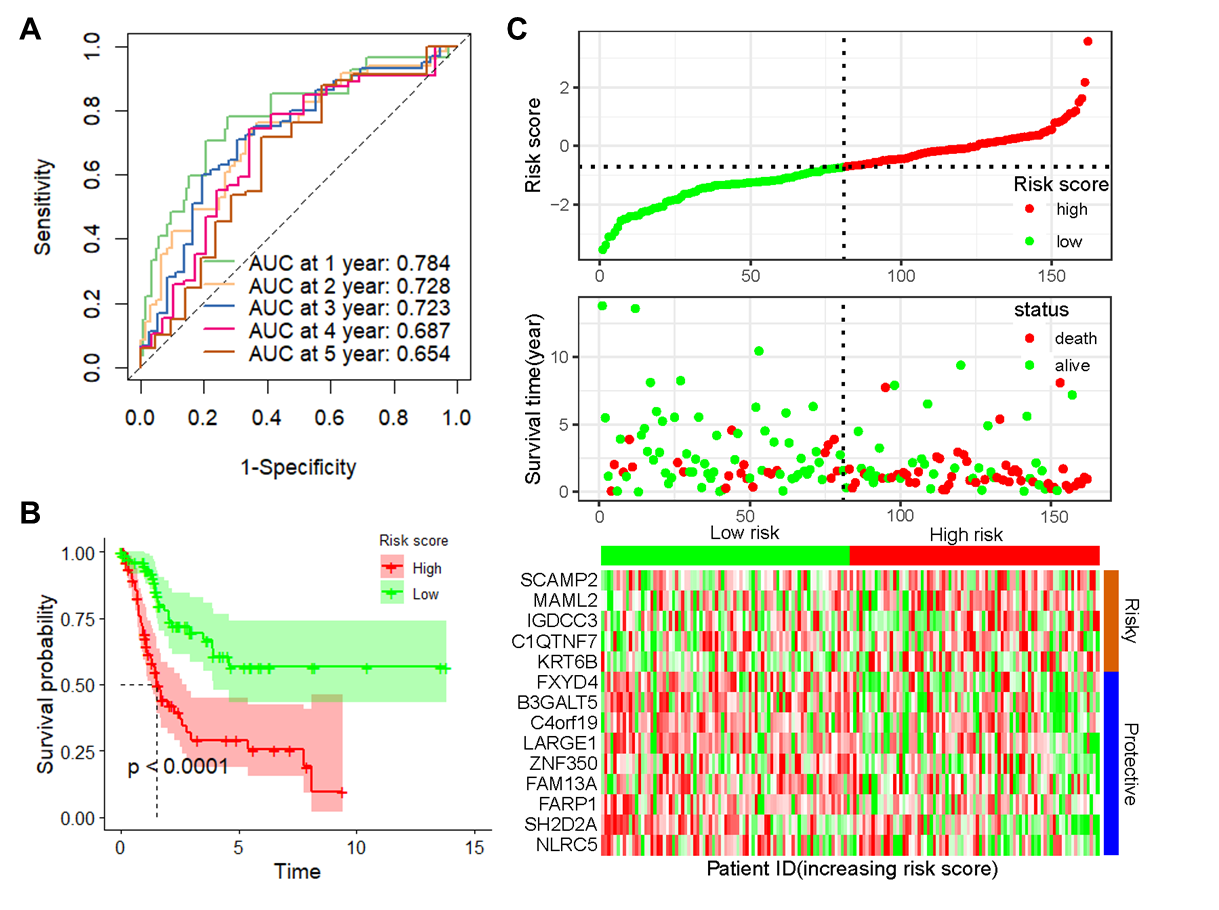
**

**Figure S2.** Validation of the prognostic model with 14 CSRGs constructed by training dataset in internal testing set. ROC curve (A) and Kaplan–Meier curve (B) for overall survival in internal testing dataset. (C) Risk score distribution, survival status and the expression of 14 CSRGs in internal testing dataset. CSRGs: cisplatin sensitivity-related genes. ROC: receiver operating characteristic curve.

**
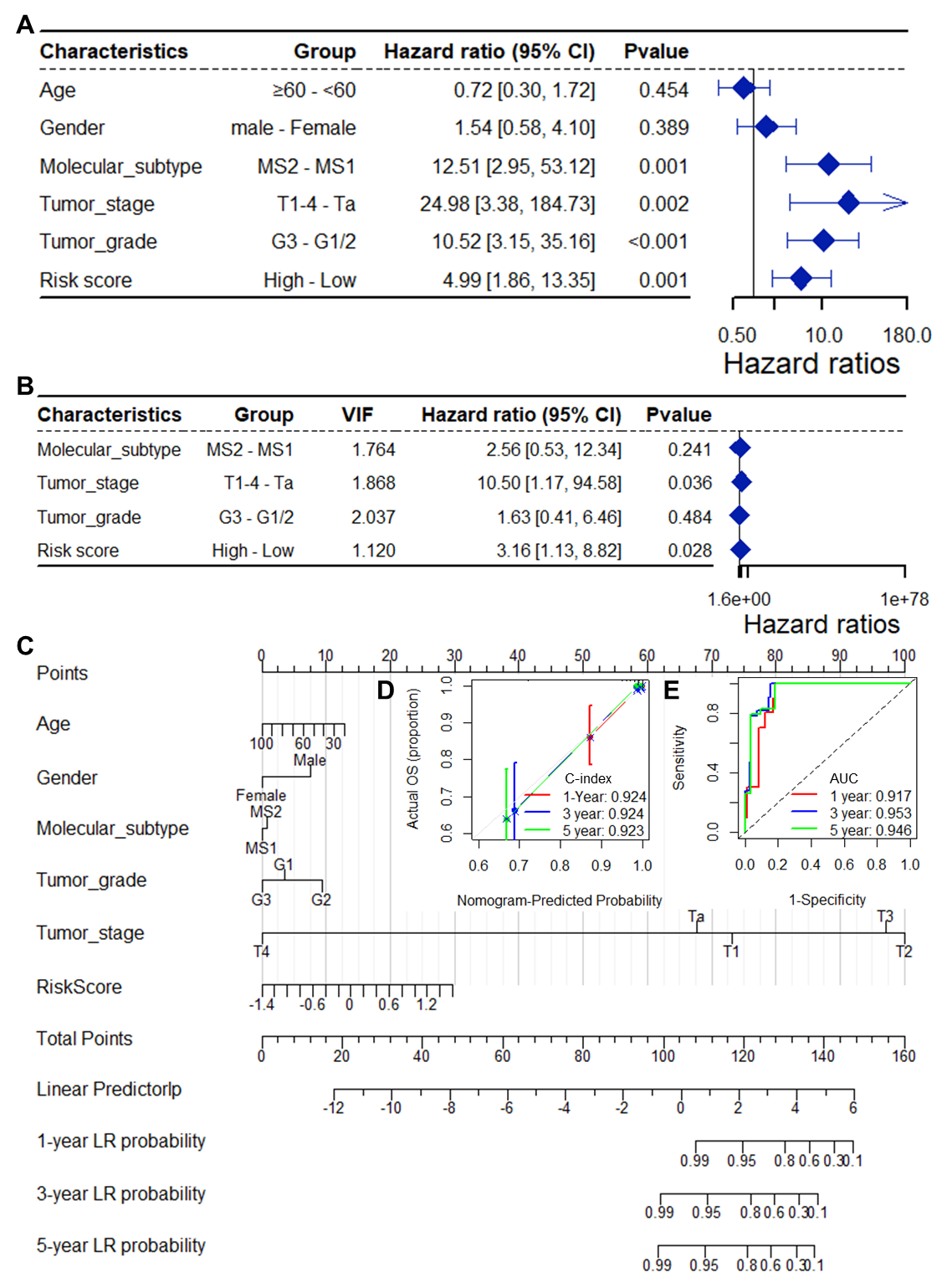
**

**Figure S3.** The CSRGs risk score was an independent prognostic factor for OS in the GSE32894 dataset. Univariate (A) and multivariate (B) Cox regression analyses of the risk score and clinicopathological features for overall survival in the GSE32894 dataset. (C) The nomogram consists of the 14-gene risk score and 6 clinical indicators based on the GSE32894 dataset. The points from these variables are combined, and the locations of the total points are determined. The total points projected on the bottom scales indicate the probabilities of 1-year, 3-year and 5-year overall survival. Calibration plots (D) and receiver operating characteristic (ROC) curves (E) were used to validate the prognostic nomogram constructed based on the TCGA- BLCA dataset. CSRGs: cisplatin sensitivity-related genes. ROC: dependent receiver operating curve. TCGA, the cancer genome map. BLCA: bladder cancer.


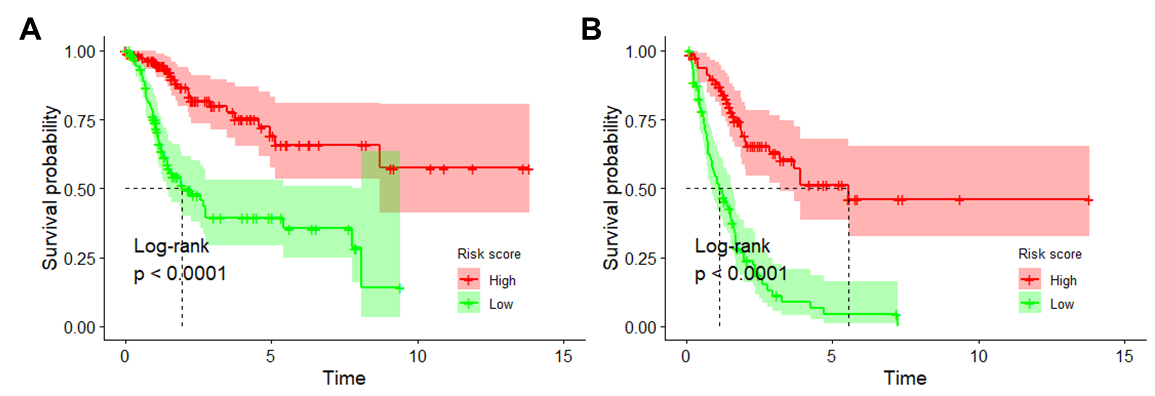


**Figure S4.**Survival curves for patients with different pathological T stages. (A) Survival curve for patients with T1/T2 stages. (B) Survival curve for patients with T3/T4 stages.

**
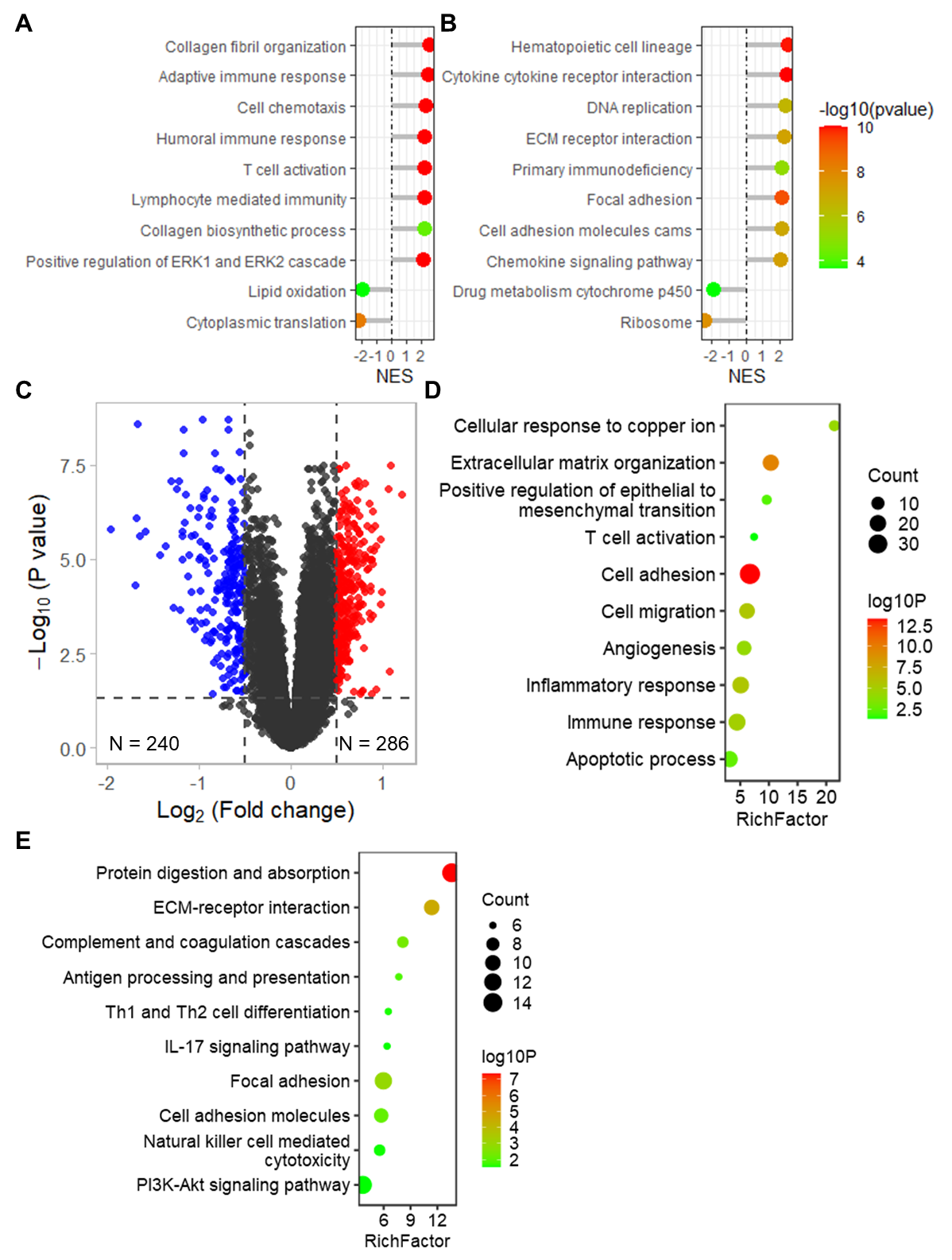
**

**Figure S5.** Enrichment analysis revealed that the risk score links to biological functions and pathways. Lollipop plots show the GSEA enrichment analysis of risk score for biological processes (A) and KEGG pathways (B). (C) Volcano plot shows the differentially expressed genes between high and low risk groups in GSE32894 dataset. Lollipop plots show the enrichment analysis of the differentially expressed genes for biological processes (D) and KEGG pathways (E). GSEA, Gene Set Enrichment Analysis. KEGG, Kyoto Encyclopedia of Genes and Genomes.

**
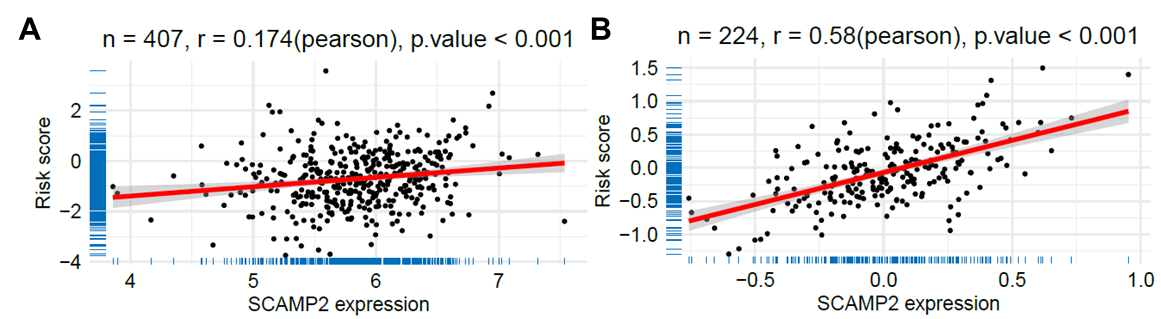
**

**Figure S6.** SCAMP is a key gene in the model. The scatter plot shows the correlation between SCAMP expression and risk score in TCGA-BLCA (A) and GSE32894 (B).

**Figure S7.** Detection of SCAMP expression in bladder cancer cells with overexpression or knockdown of SCAMP by qPCR.
